# A model of the levator-depressor neuro-mechanical system of the stick insect leg

**DOI:** 10.1186/1471-2202-12-S1-P337

**Published:** 2011-07-18

**Authors:** Silvia Daun-Gruhn, Tibor I Toth

**Affiliations:** 1 Emmy Noether Research Group of Computational Biology, Department of Animal Physiology, Institute of Zoology, University of Cologne, D-50674 Köln, Germany

## 

It has been established in experiments that, in the stick insect, antagonistic muscle pairs of each leg joint are associated with their own central pattern generator (CPG) which is, in essence, responsible for driving the motoneurones (MNs) that innervate the muscles of the antagonistic muscle pair [1]. This arrangement ensures large flexibility of the leg movements in these animals. It is therefore of paramount interest to study the function of the CPG-MN-muscle systems at the different joints of the leg. Modelling can be a useful integrative tool in this study and in understanding the interactions between the elements (CPG, MN and muscles) of the neuro-mechanical system in question. In this paper, we report on the results of our modelling work of the levator-depressor neuro-mechanical system.

The model includes all three elements of the system: the CPG, the MNs and the levator and depressor muscles. The CPG consists of two mutually inhibitory neurones which form a relaxation oscillator [2], and which control the MN activity via inhibitory interneurones like in [3]. However, additional interneurones convey the afferent information from the campaniform sensilla (CS) to the CPG. The core of the mechanical model is the mechanical equation of motion of the femur. The muscles are modelled as nonlinear springs with variable elasticity moduli and with viscous damping parallel to the springs. Finally, the coupling between the neuronal and the muscle system is done by using a simple, linear, 1^st^ order synapse model [4]. The synaptic activity, as it varied in time, depending on the activity of the MNs, set the actual value of the elasticity moduli of the muscles (springs).

The model successfully reproduced the electrical activity of the CPG neurones and MNs, as well as the angular movement of the femur as seen during normal straightforward locomotion. In this case, the activity of the MNs was fully controlled by that of the CPG. However, additional modulation of the synaptic input to the MNs changed this picture and the angular motion of the femur could be modified to have either the depression or the levation as the dominating state, e.g. mimicking backward or curve walking. We could also simulate the experimental conditions after partial removal of the femoral CS. In this case, the original dominance of the depression was overturned, and the levator MN was continuously active because the corresponding CPG neurone showed tonic activity. However, the depressor MN and muscle could be activated in the model by stimulation of the CS. The balance between contraction of the levator and depressor muscle could again be shifted by separately modulating the synaptic input to the MNs.

In conclusion, we showed that the model could successfully mimic the behaviour of its biological counterpart.

The above simulations provided strong evidence for a high behavioural flexibility of the model under various peripheral (CS stimuli) or central (modulatory signals) influences. Some of these behavioural modes also hint at corresponding processes that might take place in the stick insect during locomotion in a changing environment.
